# Weekend–Weekday Differences in Adherence to the Mediterranean Diet among Spanish University Students

**DOI:** 10.3390/nu14142811

**Published:** 2022-07-08

**Authors:** Luis M. Béjar

**Affiliations:** Department of Preventive Medicine and Public Health, School of Medicine, University of Seville, 41004 Seville, Spain; lmbprado@us.es; Tel.: +34-954-551-771

**Keywords:** Mediterranean diet, food, feeding behavior, nutrition assessment, university, smartphone, mobile applications, information technology

## Abstract

Daily routines may influence eating patterns; however, differences in intake on weekdays and at weekends have rarely been explored. Furthermore, these differences have not been analyzed among university students (a particularly interesting group among the younger generations). The aim of the study was to evaluate weekend–weekday variation in the Mediterranean diet among Spanish university students, while investigating the potential influence of age, gender, studies, body mass index, smoking status and physical activity status. A repeated-measurement 28-day cross-sectional observational study with self-reported dietary intake collected using the e12HR app was conducted. There were 361 participants: average age 20.6 years; 72.9% women; 58.2% students of Pharmacy; average BMI 21.9 kg/m^2^; 91.4% nonsmokers; 77.6% performed ≥150 min/week of physical activity. Outcome measurements were adherence to the Mediterranean diet (AMD) index and percentage of participants meeting recommendations for each food group on weekdays and at weekends. In all subgroups, Spanish university students’ global diet was associated with low AMD, with poorer diet quality (>12% reductions in mean scores of AMD index and >26% reductions in adequate adherence scores (≥9)) at weekends. In conclusion, weekend health behaviors of Spanish university students displayed less favorable eating behavior, making the weekend an important target for public health interventions aiming to improve dietary intake.

## 1. Introduction

In 2004, the World Health Organization (WHO) adopted the Global Strategy on Diet, Physical Activity and Health, the overall goal of which was to promote and protect health through diet and physical activity [1]. Since then, improving diet quality has been essential for health promotion strategy.

Within the international debate on changing diets and food systems to more adequate ones, the Mediterranean diet (MD) is potentially the best diet based on evidence as it is at once healthy and sustainable. The MD combines: 1. Important health benefits: a growing body of evidence supports the protective role of the MD, in terms of primary and secondary prevention, against cardiovascular disease, arteriosclerosis, cancer, diabetes mellitus, metabolic syndrome, excess weight/obesity, respiratory disease (asthma and sleep apnea), mental disorders (cognitive decline and depression) and renal disease [2,3,4,5,6]. Non-nutritional aspects, linked in one way or another to food consumption, have been suggested to contribute to the beneficial effect of the MD; these include, among others, physical activity [7]; and 2. Sustainability [6,8,9,10,11,12,13]: an aspect which has been under debate in recent years regarding dietary patterns is that they should not only be beneficial for all people but also for the environment, for all countries and, as such, for the planet [6,8]. The MD does this as it has a low environmental impact and richness of biodiversity, a high sociocultural value is placed on the foods, and it provides positive local economic benefits [6].

The MD (with its health benefits and sustainability) is faced with great difficulties in its implementation in other geographic and cultural regions and, paradoxically, it is even struggling to stay alive in traditionally Mediterranean regions (influenced by unhealthy dietary habits as a result of global acculturation) [4] where it is being abandoned, mainly by younger generations [12].

People’s day to day lives are punctuated by work, education, domestic chores, sleep and food. Alterations in daily patterns contribute significantly to changes in dietary patterns [14]. Characterization of food consumption across the days of the week can help target health promotion initiatives that are striving to improve dietary intake of the population [15]. However, while much research has focused on the identification of dietary patterns, less attention has been paid to temporal variation of dietary intake [15].

A limited number of studies have analyzed weekly variation dietary patterns in diverse populations (e.g., children/adolescents [15,16,17,18,19,20,21,22,23,24,25,26,27,28,29,30,31] and adults [15,29,30,31,32,33,34,35,36,37]) residing in different countries (e.g., United States [16,17,18,19,26,29,32,33,34,36,37], Denmark [15,20,21], Australia [25,28], Canada [31,35], Brazil [30], New Zealand [22], France [24], Korea [27], Belgium, Cyprus, Estonia, Germany, Hungary, Italy, Spain and Sweden (multicentric study) [23]).

In general, findings have previously reported less healthful dietary intakes at weekends in comparison to those at weekdays. Compared with weekdays, weekend days have shown higher energy intake [15,20,29,30,32,33,34,35], higher percentage of energy from fat [16,17,29,30,32,34], higher consumption of added sugar [15,23], alcohol [15,32,34,35], discretionary foods [15,25,32], sugar-sweetened beverages [15,20,21,22,30,32], sweets [20], chocolate [20], white bread [20], and hot chips [22,30], lower consumption of dietary fiber [15,32], vegetables [15,17,20,21,24,32,34], fruit [15,16,17,20,21,24,32,34], milk/yogurt [24,34] and wholegrain products [15,20,24,34], a consistent pattern of more frequent consumption and larger portions of unhealthy foods and beverages [19], and an increase in the prevalence of fast-food and full-service restaurant consumption [32]. Additionally, some studies have provided lower dietary quality scores on weekends than on weekdays, such as the Dietary Guidelines Index for Children and Adolescents score [25] and the Healthy Eating Index [34,35]. However, the nature of weekday–weekend variation in dietary intake among university students (a particularly interesting group among the younger generations) has remained unknown.

The university phase may be the first phase of life when most teenagers start making their own food choices. For this reason, universities may provide an ideal forum for reaching out to many young adults through nutrition education programs that may positively influence students’ eating habits [38]. Research on the timing of dietary behaviors is essential for understanding the complexity of dietary patterns in this novel group and necessary to inform nutrition-related health policies and recommendations, which, regarding the MD, could help reverse the abandonment observed especially in the younger generations.

Therefore, to our knowledge, this paper serves as the first attempt to evaluate weekend–weekday differences in the MD among Spanish university students through 28 days of dietary recording, while investigating the potential influence of age, gender, studies, body mass index (BMI), smoking status and physical activity status as determinants for weekly variation. We hypothesized that individuals would display a less healthy diet during the weekends compared with that of the weekdays and furthermore that these differences were heterogeneous across population subgroups under examination.

## 2. Materials and Methods

### 2.1. Participant Recruitment

A repeated-measurement 28-day cross-sectional observational study was conducted. The recruitment of participants took place in April 2022.

The study took place with students at the Faculties of Medicine and Pharmacy at the University of Seville (Andalusia, Spain, South of Europe), selecting 4 random classrooms in each school.

A member of the research team presented the project to the students providing information on:

1. The objectives and participation in the study: to participate, interested students had to send an email to the address provided for the study;
2. Inclusion criteria: over the age of 18; no chronic pathologies, food intolerances or pregnancy (situations that could require specialized dietary recommendations); be a student at the Faculties of Medicine or Pharmacy (University of Seville); and possess a mobile telephone with Internet access and an iOS or Android operating system;
3. How the e-12HR app works.

After receiving an e-mail from the interested students, a member of the research team responded with another e-mail that contained the following documents and information:

1. Informed consent: the student had to sign and return it to the same e-mail address;
2. An initial document with personal information (date of birth, gender, center of study, height, weight, smoking status): the student must complete and return to the same e-mail address;
3. Their assigned personal alphanumeric code;
4. The method for downloading the e-12HR app: the app is free to download in the Apple Store (for iOS operating systems) or the Play Store (for devices with the Android operating system);
5. A user manual with detailed information for using the e-12HR app.

This procedure was implemented to promote participation in the study, avoiding unnecessary travel in order to sign or fill in documents, as well as to avoid the unnecessary use of paper and to promote conservation.

Participation in the study was incentivized with a raffle for school materials (valued at 500 euros) among the participants who completed the study.

### 2.2. e-12HR App

e-12HR is a previously validated application that allows for long-term collection of data on dietary intake of food groups [39,40,41,42,43].

After downloading, the first time that a participant used the e-12HR mobile application, they had to activate it by introducing a personally assigned alphanumeric code. After this step, the participants registered the number of standard portions consumed during the day for each of the 18 food groups included in the study: fruits, vegetables, breakfast cereals, pasta, rice, bread, olive oil, milk and dairy products, nuts, fermented beverages (wine and beer), potatoes, legumes, eggs, fish, white meat, red meat and sweets. The application also allowed users to register the number of minutes of moderate and intense physical activity performed throughout the day. 

The participants were instructed to use the app after finishing their last meal of the day [44,45]. The app could only be completed between 8PM and 4AM, a time range that might seem strange at first glance but was chosen to provide users with sufficient time to complete the task. Taking into account that the university students, young adults, who made up the sample often go out in the evening and eat and drink until late at night, this time period allowed users to also register those foods/drinks in the application. 

At the end of each day of monitoring, an alert appeared on the mobile phone of the participant to let them know it was time to use the app (each participant was allowed to establish the time for the alert according to their own preference). From that moment on, the participant could access the task and register the number of standard servings consumed throughout the day for each of the previously mentioned food groups, and the number of minutes of physical activity. 

**Figure 1:**
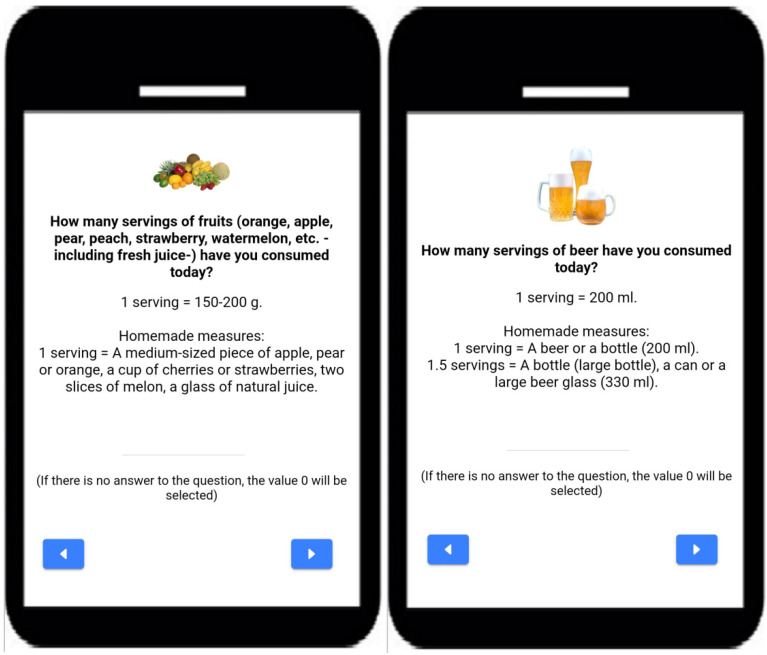
Screenshots from the e-12HR app.

In order to assist in estimating the number of standard servings consumed, each food group was accompanied by an explanatory text with different homemade measurements (as the research team considered that it would be more appropriate and easier to follow for people without experience in dietetics). The standard servings used by e-12HR are based on a semiquantitative food frequency questionnaire (FFQ) previously validated for the Spanish population [46]. For example, when using the app, participants would see the following: How many servings of fruits (orange, apple, pear, peach, strawberry, watermelon, etc., including fresh juice) have you consumed today? One serving = 150–200 g. Homemade measures: 1 serving = A medium-sized piece of apple, pear or orange, a cup of cherries or strawberries, two slices of melon, a glass of natural juice. Participants would introduce the corresponding number in the “Answer” section and then they would tap the “Next” button to continue on to the following food group. The app also allowed participants to use decimals to better estimate the number of portions consumed. If an error occurred when registering information, participants could return to the previous page by tapping the “Previous” button, and they could begin the process again (Figure 1).

To make the app easier to use, the list of food groups appeared in the same order every day (Appendix A, Table A1) and each food group was accompanied by a representative image.

After completing the daily questionnaire on the app, the collected information was automatically sent to the website of the study administrators, so the participants could not change their previous responses nor access the application until the following day when they would complete the next questionnaire.

Registration of dietary data using the e-12HR app was scheduled for twenty-eight consecutive days. Participants could know if they had completed the study period as the application presented a counter with the number of days the task was successfully completed.

### 2.3. Usability Rating Questionnaire for e-12HR

Finally, at the end of the 28-day study period, a member of the research team sent an e-mail to each participant that contained a usability rating questionnaire [43,47,48,49] for the e-12HR app, comprised of five questions about the completion the daily e-12HR task (Appendix A, Table A2).

Each participant was required to complete and return this usability rating questionnaire to the same e-mail address.

### 2.4. Adherence to Mediterranean Diet (AMD) Assessment

To calculate the adherence to Mediterranean diet (AMD) index, it took into account previously established rules [50] which consider:

1. Specific food groups (compatible with the MD);
2. Recommendations for consumption frequency for standard servings (per meal, daily or weekly);
3. A numerical score assigned to each item (Appendix A, Table A3).

However, it was necessary to make some modifications in order to adapt them to the characteristics of e-12HR (Table 1).

For food groups that have scores greater than 1, the scoring rules are as follows [51]:

1. Fruits contribute 1 point for 1 to 2 servings, 2 points for 2 to 3 servings, and 3 points for 3–6 servings per day.
2. Vegetables contribute 1 point for 2 to 4 servings, 2 points for 4 to 6 servings, and 3 points for ≥6 servings per day.
3. Cereals contribute 1 point for 1 to 2 servings, 2 points for 2 to 3 servings, and 3 points for 3–6 servings per day.
4. Olive oil contributes 1 point for 1 to 2 serving, 2 points for 2 to 3 servings, and 3 points for 3–4 servings per day.
5. Milk and dairy products contribute 1 point for 1 to 2 servings and 2 points for 2–3 servings per day.
6. Nuts contribute 2 points for 1–2 servings per day.

For each food group, if the indicated recommendations were not followed, a value of zero was assigned for that group.

Scorings for the AMD index were calculated manually by the research team (using the data sent by the application to the study website and the rules shown in Table 1).

The information from each participant was analyzed considering three time periods: 1. Total monitoring period; 2. Weekdays (Monday–Thursday); and 3. Weekend days (Friday–Sunday and holidays).

For each period of time, for each individual food group, the average daily number of standard portions registered throughout the period in question was first added all together, and the result was then divided by the number of days for which the task was completed during that same period. For each period of time, for food groups which used the weekly averages for the number of standard servings, as opposed to daily, the result from the previous operation was multiplied by 7.

Obvious errors produced during data entry were modified (as it was considered that the data must have been introduced as grams or milliliters instead of standard servings). For example, on one occasion, a value of 150 was introduced for the question “How many servings of vegetables (tomato, carrot, bell pepper, lettuce, zucchini, etc.) have you consumed today?”. The research team considered that this value indicated a consumption of 150 g, which is the equivalent of one serving. In any case, the data were modified by the research team on only 1080 occasions out of a total of 196,452 registered data points (0.55%).

To complete the process, all of the values were added up, and scoring of the AMD index was generated, which could vary between zero and twenty-four.

On top of this, the score on the AMD index was related with one of three levels of AMD [51]: low (0–8 points), moderate (9–15 points) or high (16–24 points).

### 2.5. Statistical Analysis

Discrete variables are presented as a number followed by percentages. Continuous variables are presented using means and standard deviations and median and interquartile range (IQR).

The data rwee tested for normality using the nonparametric Kolmogorov–Smirnov test.

For unpaired samples, Students *t*-test or the nonparametric Mann–Whitney U-test were used for the analysis of quantitative variables, and the chi-square test was used for the comparison of proportions.

For paired samples, Students *t*-test or the nonparametric Wilcoxon test were used for the analysis of quantitative variables, and McNemar’s test was used for the comparison of proportions.

The results were considered significant if *p*-value < 0.05.

Statistical analyses were performed using the SPSS statistical software package version 26.0 (SPSS Inc., Chicago, IL, USA).

## 3. Results

### 3.1. Database

The informed consent forms were signed by 439 students who fulfilled the inclusion requirement for the study. Of them, 78 were considered as nonresponsive (as they completed the task on the app for less than 14 days); the data for these individuals was not included in the later statistical analysis. There were no significant statistical differences in the variables studied between the participants who completed the study and those who did not. The study response rate was 82.2% (361 of 439 students completed the task on the app for at least 14 days).

As has been mentioned previously, dietary data registration through the e-12HR app was scheduled for twenty-eight consecutive days. The majority of the responsive participants, 79.2%, completed the monitoring period and of note, more than a third of them, 39.6%, continued using the app for more than thirteen days on their own initiative. The vast majority of the participants completed the application at least seven days both on weekdays and weekend days. The information on the number of days for which the task was completed is shown in Table 2.

The average age of the participants was 20.6 years old. The gender distribution of women/men was 72.9%/27.1%. More than half of them were students at the School of Pharmacy, 58.2%. The average BMI was 21.9 kg/m^2^. The majority of the participants were nonsmokers, 91.4%, and performed 150 min or more of moderate/intense physical activity each week, 77.6% (Table 2).

The participants who completed the study registered their daily consumption for the 18 food groups included in the study for 10,914 days taken together (representing a collected total of 196,452 data points on daily consumption for the food groups).

### 3.2. Scores and Levels of the AMD Index

Table 3 shows the scores and levels of the AMD index in the total monitoring period in the whole study sample and in different subgroups thereof (by age, gender, studies, BMI, smoking status and physical activity status). The mean score of the AMD index was 8.7 (SD = 2.9), by subgroups varying between 7.6 (<150 min/week) and 9.2 (males). There were statistically significant differences in the subgroups by gender, BMI and physical activity status: adherence scores were higher in subgroups of males (9.2), <25 kg/m^2^ (8.8) and ≥150 min/week (9.0), respectively. An adequate adherence score (≥9) was observed in 47.9% of the participants (although of them, only 1.9% (seven subjects) presented a high level (16–24 points)). By subgroups, the lowest percentage of an adequate AMD score was found among <150 min/week (30.9%), and the highest percentage among ≥150 min/week (52.9%). There was a statistically significant difference in the subgroups by physical activity status.

Table 4 and Table 5 report the scores and levels of the AMD index on weekdays and weekend days. The differences were statistically significant, considering the scores and levels of the AMD index, in the whole study sample and in all subgroups thereof (except in smoking status yes for the level of the AMD index).

Compared with the weekday average, the Mediterranean diet Serving Score (MDSS) for the weekend average was 1.2 points lower (12.6% reduction). By subgroups, the reduction varied between 18.1% (smoking status yes) and 12.0% (smoking status no) (Table 4).

Compared with that of the weekdays, the adequate adherence score (≥9) for weekends was 17.8 percentage points lower (31.8% reduction). By subgroups, the reduction varied between 26.3% (≥20 years) and 41.2% (<150 min/week) (Table 5).

### 3.3. Stratification of AMD Items (Food Groups)

Table 6 shows the stratification of AMD items (food groups) in the total monitoring period. Fruits, vegetables, cereals, olive oil, milk and dairy products and nuts (food groups which contribute the highest scores to the MDSS index, Table 1) were consumed according to recommendations by less than 25% of the participants, in the whole study sample and in all subgroups thereof (except for cereals in males, milk and dairy products in smoking status yes and physical activity status ≥150 min/week). Other food groups that were also consumed according to the recommendations by less than 25% of the participants were red meat (in the whole study sample and in all subgroups thereof), sweets (in subgroups <20 years, females, BMI ≥25 kg/m^2^, smoking status yes and physical activity status <150 min/week) and legumes (in smoking status yes). There were statistically significant differences in the subgroups by gender (eight food groups), physical activity status (five food groups), age and smoking status (two food groups), studies and BMI (one food group).

Table 7 reports the stratification of AMD items (food groups) on the weekdays and weekend days. The consumption on weekends was similar to that on the weekdays in the whole study sample and in all subgroups thereof for the food groups which contribute the highest scores to the MDSS index (fruits, vegetables, cereals, olive oil and nuts (except for olive oil and nuts among student of Medicine)) and fish. A lower percentage of participants complied with the recommendations in consumption at weekends than on weekdays in sweets (in the whole study sample and in all subgroups), fermented beverages (in the whole study sample and 11 subgroups), potatoes and legumes (in the whole study sample and 10 subgroups), milk and dairy products (in the whole study sample and eight subgroups), red meat (in the whole study sample and six subgroups), eggs (in the whole study sample and five subgroups) and white meat (in the whole study sample and three subgroups).

### 3.4. Usability Rating Questionnaire for e-12HR

The usability rating questionnaire was answered by 174 participants. The responses of the users are shown in Table 8.

## 4. Discussion

To our knowledge, this study is the first to evaluate weekend–weekday differences in diet among university students (specifically, MD and Spanish university students at the Faculties of Medicine and Pharmacy). As we hypothesized, dietary intake in this sample was found to fluctuate during the week, with weekend days displaying lower dietary quality compared with that of weekdays; however, the differences were very similar across the population subgroups under examination.

A rationale for the existence of distinct, temporally conditioned behavioral patterns, as observed in this study, could be provided by the fact that human behavior during the weekdays is highly structured and generally dictated by time spent at work/in school [14], and many health promotion initiatives target the weekdays. A change in daily structures during the weekend is likely to have consequences for habitual behaviors such as eating [15].

Spanish university students are a particularly interesting group among the younger generations for various reasons. University students are one of the populations with a high risk of having unhealthy habits [52]; generally, young adults, especially university students, present an important challenge due to the coincidence of a series of emotional, physiological and environmental changes. They select their food, are very receptive to fashionable trends such as following slimming diets, skipping meals or consuming snacks, soft drinks and other new products [53], and leading an unhealthy lifestyle, and the risk that these habits are preserved during adulthood represents a great inconvenience for these individuals, especially for students in fields related to Health Sciences [54]. The promotion of healthy habits in the population at large, such as maintaining a good diet, is one of the primary tasks of these future professionals. Therefore, they must not only know the basics of these habits, but also practice them [55].

Spanish university students at the Faculties of Medicine and Pharmacy (this study sample) have compulsory and optional subjects related to nutrition and dietetics: for students of Medicine, the subjects Preventive Medicine and Public Health (compulsory) and Health Promotion (optional); for Pharmacy students, the subjects Public Health (compulsory), Nutrition and Bromatology (compulsory) and Nutrition, Dietetics and Dietotherapy (optional). This training in nutrition/dietetics may have had an influence on the dietary habits of the participants. For this reason, future research will focus on determining weekend–weekday differences in diet among other non-Health Science students (without training in health or nutrition) (see future research related to the current study section).

In general, in the present study, in the whole study sample and in all subgroups thereof (by age, gender, studies, BMI, smoking status and physical activity status): A. In the total monitoring period: Adherence to the MD (by mean scores of the AMD index and adequate adherence score (≥9)) was low (Table 3). This is similar to previous recent literature among Spanish university students (although using other AMD indexes) [56,57,58,59]. Among the categories, gender, BMI and physical activity status provided statistically significant differences (although physical activity status was the only one to show any statistically significant differences, in both the AMD index and adequate adherence score (≥9); gender and BMI only in the AMD index) (Table 3). The AMD index was higher in males compared to that in females, which was a finding that follows the trend shown in these recent studies [58,59]. Also in line with one of these previous studies was that maintaining low levels of physical activity was associated with a diet of worse quality [56]. In contrast, in these recent papers among Spanish university students, the relationship of a low score of the AMD index with overweight/obesity could not be observed [56,58,59]. The food groups which were consumed according to recommendations by less than 25% of the participants were fruits, vegetables, cereals, olive oil, milk and dairy products, nuts, red meat and sweets (Table 6). B. During weekend days, compared with the weekdays: Poorer diet quality was observed. This study confirmed findings from previous research in children/adolescents [15,16,17,18,19,20,21,22,23,24,25,26,27,28,29,30,31] and adults (not specifically university students) [15,29,30,31,32,33,34,35,36,37]: substantial reductions in following dietary guidelines were observed, with >12% reductions in mean scores of the AMD index and >26% reductions in adequate adherence scores (≥9) (Table 4 and Table 5). There was no percentage variation in consumption of fruits, vegetables, cereals, olive oil, nuts and fish (Table 7). There was a lower percentage of participants who complied with the recommendations in consumption of sweets, fermented beverages, potatoes, legumes, milk and dairy products, red meat, eggs and white meat (Table 7).

The Spanish university students’ diet was associated with poor diet quality in general and, especially at weekends, in the whole study sample and in all subgroups thereof. Among Spanish university students, policy interventions and public health campaigns are warranted to promote healthy eating focused on all time periods, on food groups that were consumed according to recommendations by less than 25% of the participants (fruits, vegetables, cereals, olive oil, milk and dairy products, nuts, red meat and sweets). In these food groups, the percentage of university students that meets the recommendations was below 25% and, additionally, no differences were observed between the weekends and the weekdays. These food groups (except red meat and sweets) contribute the highest scores to the MDSS index (Table 1), so an improvement in their consumption would be associated with notable increases in the AMD index. At the same time, focus should be placed on the weekends and food groups with a lower percentage of participants which complied with the consumption recommendations (sweets, fermented beverages, potatoes, legumes, milk and dairy products, red meat, eggs and white meat). An improvement in their consumption at the weekends could be associated with increases in the global AMD index. In this sense, future interventions should be applied to all subgroups by age, gender, studies, BMI, smoking status and physical activity status (with very similar results across subgroups under examination).

Only 174 of 361 (48.2%) participants answered the usability rating questionnaire. Regarding this point, on the one hand, the recruitment of the participants took place in April 2022. On the other, the monitoring period was 28 days, and only at the end of these 28 days of study was the email sent to the participants (with the usability rating questionnaire). Taking into account the participant recruitment period (April 2022) and the duration of the study (28 days), the email was received by the participants from the beginning of May onwards, close to the second semester exams. This fact could have influenced the low response rate to the usability rating questionnaire, since the students were already focused on preparing for exams at that time.

The strengths of this study include, among others, the 28-day dietary intake data collection period for each participant. While those participants who completed the app task for at least 14 days were considered responders, the majority of the participants repeated the task for at least 28 days, (Table 2). The decision to use the 14-day limit was due to other studies that included repeated dietary intake data collections for 14 days or less (2 days [25,26,29,30,32,37], 3 days [17,24,28], 5 days [33], 7 days [15,16,20,21], 14 days [18]), except for a single study that included a larger number of repetitions, specifically 52 repetitions in total [34]. In order to determine habitual dietary intake (or average long-term consumption) using short-term tools, it is necessary to repeat these measures multiple times, and more repetitions provide a clearer picture of habitual dietary intake. This has been possible thanks to the use of e-12HR. This app is, basically, a modified 24 h recall (24HR) which is completed once a day during the study period and does not require making photographs of the food groups consumed [39,40,41,42,43]. These characteristics result in a low workload for users of e-12HR and, at the same time, has allowed the research team to schedule a longer monitoring period. The majority of the participants in this study reported that the e-12HR app was easy and interesting to complete and contained understandable questions; that they would be willing to complete e-12HR app again; and that the task took 3 min or less per day to complete (Table 8); Another important strength of the study was the various subgroups that were analyzed (age, gender, studies, BMI, smoking status and physical activity status).

Nevertheless, a few limitations should be noted. e-12HR is a self-reporting method and, as a consequence, it presents the limitations inherent in this type of tool (amply described in [47,60,61,62,63,64,65]); among others, the dependence on the memory of each participant and, mainly, the difficulty of estimating the size of servings consumed. As far as the participant’s memory is concerned, e-12HR reduces this disadvantage, as it only requires short-term memory, being completed at the end of each day. Indeed, the name e-12HR (electronic 12-Hour Dietary Recall) is a reference to how it differs from a 24HR. While 24HR registers consumption of food groups from the day before, e-12HR allows users to register their consumption during the same day (normally with a maximum period of time between consumption of the food and it being registered of 12 h). Regarding the difficulty of estimating the size of the servings consumed, we must take into account that AMD assessment does not require a precise estimation of the size of the foods consumed, but rather a precise recognition of the food groups consumed together with an approximate estimation of the servings [51]. In this sense, e-12HR allows for collecting data on consumption for all the food groups needed to calculate AMD. Newer alternative methods for determining dietary intake include audio signal processing, inertial sensing, image processing, non-intrusive near-infrared scanning and gesture recognition interfacing [66,67,68,69]. Some authors maintain that more research is needed to develop these and other tools, which are more objective and precise, and that resources should be invested to this end [65]. Until these alternatives are available and in spite of their limitations, digital technologies for self-reporting methods can, and must, be developed and utilized [39] as an improvement on traditional self-reporting methods, the progress of which constitutes one of the most important challenges to the field of nutritional epidemiology [48,70] in this day and age. The small number of individuals in some of the subgroups analyzed (e.g., smoking status yes (n = 31)) is another of the limitations of the study.

### Future Research Related to the Current Study

Future research will focus on determining weekend–weekday differences in diet among Health Science students during nonschool periods as well as among other students (non-Health Science students). This will allow the research team to establish if the results observed in the present study are specific to Health Science students and to the school period or if, on the contrary, they are maintained in other periods (e.g., the summer holidays) and among other students (without training in health or nutrition).

## 5. Conclusions

This study has been carried out using the e-12HR application. This app is characterized by a low workload for users (allowing the research team to schedule a long monitoring period) as it only requires short-term memory use and allows for collecting data on consumption for all the food groups needed to calculate AMD. Preliminary evidence suggests that Spanish university students’ diet was associated with poor diet quality in general and especially at the weekends in the whole study sample and in all subgroups thereof. These results could be used to improve public health campaigns to promote healthy eating among Spanish university students. In a practical way, the interventions could be focused, on the one hand, on all times periods, on food groups that were consumed according to recommendations by less than 25% of the participants (fruits, vegetables, cereals, olive oil, milk and dairy products, nuts, red meat and sweets), and on the other, on weekends, on food groups with a lower percentage of participants who complied with the consumption recommendations (sweets, fermented beverages, potatoes, legumes, milk and dairy products, red meat, eggs and white meat). Finally, future interventions should be applied to all subgroups by age, gender, studies, BMI, smoking status and physical activity status (with very similar results).

## Figures and Tables

**Table 1:** Mediterranean diet Serving Score for e-12HR.

Scoring of Food Groups Calculated on a Daily Basis		
Food Group	Servings Per Day	Score (e-12HR)
Fruits	3–6 servings	3
Vegetables	≥6 servings	3
Cereals	3–6 servings	3
Olive oil	3–4 servings	3
Milk and dairy products	2–3 servings	2
Nuts	1–2 servings	2
Fermented beverages	1–2 servings	1
**Scoring of food groups calculated on a weekly basis**		
**Food group**	**Servings per week**	**Score (e-12HR)**
Potatoes	≤3 servings	1
Legumes	≥2 servings	1
Eggs	2–4 servings	1
Fish	≥2 servings	1
White meat	2–3 servings	1
Red meat	<2 servings	1
Sweets	≤2 servings	1
Total maximum score		24

Cereals: breakfast cereals, pasta, rice and bread. Olive oil: used on salads or bread or for frying. Milk and dairy products: milk, yogurt and cheese. Fermented beverages: wine and beer. White meat: poultry. Red meat: pork, beef and lamb. Sweets: sugar, candies, pastries, sweetened fruit juices and soft drinks.

**Table 2:** Characteristics of study participants.

Characteristics	N (%)	Mean (SD)	Median (IQR)
Participants who completed the study	361 (82.2)	-*	-
Participants who did not complete the study	78 (17.8)	-	-
Number of days app task completed	-	-	-
>30 days	143 (39.6)	-	-
28–30 days	143 (39.6)	-	-
21–27 days	55 (15.2)	-	-
14–20 days	20 (5.5)	-	-
Number of days app task completed (weekdays: Monday–Thursday)	-	-	-
15–32 days	287 (79.5)	-	-
7–14 days	73 (20.2)	-	-
3–6 days	1 (0.3)	-	-
Number of days app task completed (weekend days: Friday–Sunday and holidays)	-	-	-
15–32 days	137 (37.9)	-	-
7–14 days	212 (58.7)	-	-
3–6 days	12 (3.3)	-	-
Age (years)	-	20.6 (2.8)	20.0 (2.0)
<20	185 (51.2)	-	-
≥20	176 (48.8)	-	-
Gender	-	-	-
Females	263 (72.9)	-	-
Males	98 (27.1)	-	-
Studies	-	-	-
Pharmacy	210 (58.2)	-	-
Medicine	151 (41.8)	-	-
BMI (kg/m^2^)	-	21.9 (3.3)	21.1 (4.0)
<25	317 (87.8)	-	-
≥25	44 (12.2)	-	-
Smoking status	-	-	-
No	330 (91.4)	-	-
Yes	31 (8.6)	-	-
Physical activity status (minutes/week)	-	-	-
≥150	280 (77.6)	-	-
<150	81 (22.4)	-	-

* Not applicable.

**Table 3:** Scores and levels of the AMD index (total monitoring period).

Total Monitoring Period	Index			Level		
	Mean (SD)	Median (IQR)	*p* *	Low: 0–8	Moderate–High ≥ 9	*p* **
All	8.7 (2.9)	8.0 (3.0)		188 (52.1)	173 (47.9)	
Age (years)						
<20	8.7 (2.8)	8.0 (4.0)	0.860	99 (53.5)	86 (46.5)	0.576
≥20	8.7 (2.9)	8.0 (3.0)		89 (50.6)	87 (49.4)	
Gender						
Females	8.5 (2.8)	8.0 (3.0)	0.049	141 (53.6)	122 (46.4)	0.339
Males	9.2 (3.1)	9.0 (4.0)		47 (48.0)	51 (52.0)	
Studies						
Pharmacy	8.6 (2.9)	8.0 (3.0)	0.496	113 (53.8)	97 (46.2)	0.437
Medicine	8.8 (2.8)	9.0 (4.0)		75 (49.7)	76 (50.3)	
BMI (kg/m^2^)						
<25	8.8 (2.9)	8.0 (4.0)	0.026	159 (50.2)	158 (49.8)	0.050
≥25	7.8 (2.4)	7.0 (3.0)		29 (65.9)	15 (34.1)	
Smoking status						
No	8.8 (2.9)	8.0 (3.0)	0.078	169 (51.2)	161 (48.8)	0.283
Yes	7.7 (2.6)	7.0 (3.0)		19 (61.3)	12 (38.7)	
Physical activity status (minutes/week)						
≥150	9.0 (2.9)	9.0 (4.0)	0.000	132 (47.1)	148 (52.9)	0.000
<150	7.6 (2.5)	7.0 (3.0)		56 (69.1)	25 (30.9)	

SD: standard deviation. IQR: interquartile range. * Evaluated by the Mann–Whitney U-test. ** Evaluated by the chi-square test.

**Table 4:** Scores of the AMD index (weekdays–weekends).

	Weekdays		Weekends		
	Mean (SD)	Median (IQR)	Mean (SD)	Median (IQR)	*p*
All	9.3 (3.0)	9.0 (4.0)	8.1 (2.9)	8.0 (4.0)	0.000 **
Age (years)					
<20	9.3 (3.0)	9.0 (4.0)	8.1 (3.0)	8.0 (4.0)	0.000 **
≥20	9.2 (3.0)	9.0 (4.0)	8.1 (2.8)	8.0 (4.0)	0.000 **
Gender					
Females	9.0 (2.9)	9.0 (4.0)	7.9 (2.8)	8.0 (4.0)	0.000 **
Males	9.9 (3.2)	10.0 (4.0)	8.6 (3.2)	8.0 (4.0)	0.000 **
Studies					
Pharmacy	9.1 (2.9)	9.0 (4.0)	8.0 (3.0)	8.0 (4.0)	0.000 **
Medicine	9.4 (3.0)	9.0 (4.0)	8.2 (2.8)	8.0 (3.0)	0.000 **
BMI (kg/m^2^)					
<25	9.4 (3.0)	9.0 (4.0)	8.2 (2.9)	8.0 (4.0)	0.000 **
≥25	8.5 (2.5)	8.0 (4.0)	7.4 (2.7)	7.0 (5.0)	0.001 *
Smoking status					
No	9.3 (3.0)	9.0 (4.0)	8.2 (3.0)	8.0 (4.0)	0.000 **
Yes	8.5 (2.7)	8.0 (4.0)	7.0 (2.4)	7.0 (4.0)	0.000 **
Physical activity status (minutes/week)					
≥150	9.6 (3.0)	9.0 (4.0)	8.4 (2.9)	8.0 (4.0)	0.000 **
<150	8.1 (2.4)	8.0 (4.0)	7.0 (2.7)	7.0 (4.0)	0.000 **

SD: standard deviation. IQR: interquartile range. * Evaluated by t-test. ** Evaluated by the Wilcoxon test.

**Table 5:** Levels of the AMD index (weekdays–weekends).

	Weekdays		Weekends		*p* *
	Low: 0–8	Moderate–High ≥ 9	Low: 0–8	Moderate–high ≥ 9	
	N (%)	N (%)	N (%)	N (%)	
All	159 (44.0)	202 (56.0)	223 (61.8)	138 (38.2)	0.000
Age (years)					
<20	78 (42.2)	107 (57.8)	117 (63.2)	68 (36.8)	0.000
≥20	81 (46.0)	95 (54.0)	106 (60.2)	70 (39.8)	0.000
Gender					
Females	124 (47.1)	139 (52.9)	170 (64.6)	93 (35.4)	0.000
Males	35 (35.7)	63 (64.3)	53 (54.1)	45 (45.9)	0.002
Studies					
Pharmacy	94 (44.8)	116 (55.2)	135 (64.3)	75 (35.7)	0.000
Medicine	65 (43.0)	86 (57.0)	88 (58.3)	63 (41.7)	0.001
BMI (kg/m^2^)					
<25	136 (42.9)	181 (57.1)	192 (60.6)	125 (39.4)	0.000
≥25	23 (52.3)	21 (47.7)	31 (70.5)	13 (29.5)	0.021
Smoking status					
No	141 (42.7)	189 (57.3)	200 (60.6)	130 (39.4)	0.000
Yes	18 (58.1)	13 (41.9)	23 (74.2)	8 (25.8)	0.125
Physical activity status (minutes/week)					
≥150	112 (40.0)	168 (60.0)	162 (57.9)	118 (42.1)	0.000
<150	47 (58.0)	34 (42.0)	61 (75.3)	20 (24.7)	0.007

* Evaluated by McNemar’s test.

**Table 6:** Stratification of adherence to MD items (food groups) (total monitoring period).

Total Monitoring Period	All	Age (Years)		Gender	
-	-	<20	≥20	Female	Male
Food group	N (%)	N (%)	N (%)	N (%)	N (%)
Fruits	22 (6.1)	17 (9.2)	5 (2.8) *	16 (6.1)	6 (6.1)
Vegetables	0 (0.0)	0 (0.0)	0 (0.0)	0 (0.0)	0 (0.0)
Cereals	52 (14.4)	28 (15.1)	24 (13.6)	22 (8.4)	30 (30.6) *
Olive oil	15 (4.2)	6 (3.2)	9 (5.1)	10 (3.8)	5 (5.1)
Milk and dairy products	85 (23.5)	44 (23.8)	41(23.3)	61 (23.2)	24 (24.5)
Nuts	28 (7.8)	16 (8.6)	12 (6.8)	15 (5.7)	13 (13.3) *
Fermented beverages	351 (97.2)	181 (97.8)	170 (96.6)	261 (99.2)	90 (91.8) *
Potatoes	147 (40.7)	77 (41.6)	70 (39.8)	117 (44.5)	30 (30.6) *
Legumes	116 (32.1)	54 (29.2)	62 (35.2)	75 (28.5)	41 (41.8) *
Eggs	171 (47.4)	76 (41.1)	95 (54.0) *	141 (53.6)	30 (30.6) *
Fish	230 (63.7)	124 (67.0)	106 (60.2)	161 (61.2)	69 (70.4)
White meat	306 (84.8)	156 (84.3)	150 (85.2)	218 (82.9)	88 (89.8)
Red meat	59 (16.3)	31 (16.8)	28 (15.9)	51 (19.4)	8 (8.2) *
Sweets	95 (26.3)	43 (23.2)	52 (29.5)	61 (23.2)	34 (34.7) *
**Total monitoring period**	**All**	**Studies**		**BMI (kg/m^2^)**	
**-**	**-**	**Pharmacy**	**Medicine**	**<25**	**≥25**
**Food group**	**N (%)**	**N (%)**	**N (%)**	**N (%)**	**N (%)**
Fruits	22 (6.1)	10 (4.8)	12 (7.9)	21 (6.6)	1 (2.3)
Vegetables	0 (0.0)	0 (0.0)	0 (0.0)	0 (0.0)	0 (0.0)
Cereals	52 (14.4)	26 (12.4)	26 (17.2)	45 (14.2)	7 (15.9)
Olive oil	15 (4.2)	10 (4.8)	5 (3.3)	14 (4.4)	1 (2.3)
Milk and dairy products	85 (23.5)	51 (24.3)	34 (22.5)	77 (24.3)	8 (18.2)
Nuts	28 (7.8)	15 (7.1)	13 (8.6)	26 (8.2)	2 (4.5)
Fermented beverages	351 (97.2)	202 (96.2)	149 (98.7)	309 (97.5)	42 (95.5)
Potatoes	147 (40.7)	88 (41.9)	59 (39.1)	136 (42.9)	11 (25.0) *
Legumes	116 (32.1)	66 (31.4)	50 (33.1)	105 (33.1)	11 (25.0)
Eggs	171 (47.4)	111 (52.9)	60 (39.7) ^*^	151 (47.6)	20 (45.5)
Fish	230 (63.7)	139 (66.2)	91 (60.3)	199 (62.8)	31 (70.5)
White meat	306 (84.8)	175 (83.3)	131 (86.8)	269 (84.9)	37 (84.1)
Red meat	59 (16.3)	33 (15.7)	26 (17.2)	54 (17.0)	5 (11.4)
Sweets	95 (26.3)	54 (25.7)	41 (27.2)	86 (27.1)	9 (20.5)
**Total monitoring period**	**All**	**Smoking status**		**Physical activity status (minutes/week)**	
**-**	**-**	**No**	**Yes**	**≥150**	**<150**
**Food group**	**N (%)**	**N (%)**	**N (%)**	**N (%)**	**N (%)**
Fruits	22 (6.1)	22 (6.7)	0 (0.0)	21 (7.5)	1 (1.2) *
Vegetables	0 (0.0)	0 (0.0)	0 (0.0)	0 (0.0)	0 (0.0)
Cereals	52 (14.4)	48 (14.5)	4 (12.9)	44 (15.7)	8 (9.9)
Olive oil	15 (4.2)	13 (3.9)	2 (6.5)	12 (4.3)	3 (3.7)
Milk and dairy products	85 (23.5)	77 (23.3)	8 (25.8)	74 (26.4)	11 (13.6) *
Nuts	28 (7.8)	27 (8.2)	1 (3.2)	24 (8.6)	4 (4.9)
Fermented beverages	351 (97.2)	325 (98.5)	26 (83.9)^*^	272 (97.1)	79 (97.5)
Potatoes	147 (40.7)	141 (42.7)	6 (19.4)^*^	111 (39.6)	36 (44.4)
Legumes	116 (32.1)	110 (33.3)	6 (19.4)	92 (32.9)	24 (29.6)
Eggs	171 (47.4)	153 (46.4)	18 (58.1)	122 (43.6)	49 (60.5) *
Fish	230 (63.7)	212 (64.2)	18 (58.1)	184 (65.7)	46 (56.8)
White meat	306 (84.8)	278 (84.2)	28 (90.3)	244 (87.1)	62 (76.5) *
Red meat	59 (16.3)	57 (17.3)	2 (6.5)	46 (16.4)	13 (16.0)
Sweets	95 (26.3)	89 (27.0)	6 (19.4)	81 (28.9)	14 (17.3) *

* Statistically significant differences. Evaluated by the chi-square test.

**Table 7:** Stratification of adherence to MD items (food groups) (weekdays–weekends).

	All		Age (Years)			
	-		<20		≥20	
	N (%)		N (%)		N (%)	
Food Group	Weekdays	Weekends	Weekdays	Weekends	Weekdays	Weekends
Fruits	22 (6.1)	20 (5.5)	16 (8.6)	16 (8.6)	6 (3.4)	4 (2.3)
Vegetables	1 (0.3)	0 (0.0)	1 (0.5)	0 (0.0)	0 (0.0)	0 (0.0)
Cereals	58 (16.1)	54 (15.0)	34 (18.4)	29 (15.7)	24 (13.6)	25 (14.2)
Olive oil	18 (5.0)	17 (4.7)	8 (4.3)	8 (4.3)	10 (5.7)	9 (5.1)
Milk and dairy products	98 (27.1)	76 (21.1) *	53 (28.6)	40 (21.6) *	45 (25.6)	36 (20.5) *
Nuts	31 (8.6)	28 (7.8)	18 (9.7)	16 (8.6)	13 (7.4)	12 (6.8)
Fermented beverages	355 (98.3)	335 (92.8) *	182 (98.4)	170 (91.9) *	173 (98.3)	165 (93.8) *
Potatoes	187 (51.8)	127 (35.2) *	96 (51.9)	64 (34.6) *	91 (51.7)	63 (35.8) *
Legumes	151 (41.8)	89 (24.7) *	76 (41.1)	41 (22.2) *	75 (42.6)	48 (27.3) *
Eggs	152 (42.1)	126 (34.9) *	75 (40.5)	54 (29.2) *	77 (43.8)	72 (40.9)
Fish	216 (59.8)	205 (56.8)	111 (60.0)	111 (60.0)	105 (59.7)	94 (53.4)
White meat	299 (82.8)	278 (77.0) *	156 (84.3)	146 (78.9)	143 (81.3)	132 (75.0)
Red meat	85 (23.5)	59 (16.3) *	42 (22.7)	32 (17.3)	43 (24.4)	27 (15.3) *
Sweets	145 (40.2)	70 (19.4) *	73 (39.5)	32 (17.3) *	72 (40.9)	38 (21.6) *
	**All**		**Gender**			
	**-**		**Female**		**Male**	
	**N (%)**		**N (%)**		**N (%)**	
**Food group**	**Weekdays**	**Weekends**	**Weekdays**	**Weekends**	**Weekdays**	**Weekends**
Fruits	22 (6.1)	20 (5.5)	16 (6.1)	15 (5.7)	6 (6.1)	5 (5.1)
Vegetables	1 (0.3)	0 (0.0)	1 (0.4)	0 (0.0)	0 (0.0)	0 (0.0)
Cereals	58 (16.1)	54 (15.0)	30 (11.4)	26 (9.9)	28 (28.6)	28 (28.6)
Olive oil	18 (5.0)	17 (4.7)	10 (3.8)	12 (4.6)	8 (8.2)	5 (5.1)
Milk and dairy products	98 (27.1)	76 (21.1) *	68 (25.9)	56 (21.3) ^*^	30 (30.6)	20 (20.4) *
Nuts	31 (8.6)	28 (7.8)	19 (7.2)	14 (5.3)	12 (12.2)	14 (14.3)
Fermented beverages	355 (98.3)	335 (92.8) *	263 (100.0)	251 (95.4) ^*^	92 (93.9)	84 (85.7)^*^
Potatoes	187 (51.8)	127 (35.2) *	141 (53.6)	98 (37.3) *	46 (46.9)	29 (29.6) *
Legumes	151 (41.8)	89 (24.7) *	102 (38.8)	59 (22.4) *	49 (50.0)	30 (30.6) *
Eggs	152 (42.1)	126 (34.9) *	126 (47.9)	103 (39.2) *	26 (26.5)	23 (23.5)
Fish	216 (59.8)	205 (56.8)	150 (57.0)	143 (54.4)	66 (67.3)	62 (63.3)
White meat	299 (82.8)	278 (77.0) *	212 (80.6)	198 (75.3)	87 (88.8)	80 (81.6)
Red meat	85 (23.5)	59 (16.3) *	73 (27.8)	50 (19.0) *	12 (12.2)	9 (9.2)
Sweets	145 (40.2)	70 (19.4) *	95 (36.1)	41 (15.6) *	50 (51.0)	29 (29.6) *
	**All**		**Studies**			
	**-**		**Pharmacy**		**Medicine**	
	**N (%)**		**N (%)**		**N (%)**	
**Food group**	**Weekdays**	**Weekends**	**Weekdays**	**Weekends**	**Weekdays**	**Weekends**
Fruits	22 (6.1)	20 (5.5)	9 (4.3)	9 (4.3)	13 (8.6)	11 (7.3)
Vegetables	1 (0.3)	0 (0.0)	0 (0.0)	0 (0.0)	1 (0.7)	0 (0.0)
Cereals	58 (16.1)	54 (15.0)	34 (16.2)	25 (11.9)	24 (15.9)	29 (19.2)
Olive oil	18 (5.0)	17 (4.7)	8 (3.8)	13 (6.2)	10 (6.6)	4 (2.6) *
Milk and dairy products	98 (27.1)	76 (21.1) *	57 (27.1)	43 (20.5) *	41 (27.2)	33 (21.9)
Nuts	31 (8.6)	28 (7.8)	14 (6.7)	18 (8.6)	17 (11.3)	10 (6.6) *
Fermented beverages	355 (98.3)	335 (92.8) *	205 (97.6)	193 (91.9) *	150 (99.3)	142 (94.0) *
Potatoes	187 (51.8)	127 (35.2) *	112 (53.3)	71 (33.8) *	75 (49.7)	56 (37.1) *
Legumes	151 (41.8)	89 (24.7)*	87 (41.4)	49 (23.3) *	64 (42.4)	40 (26.5) *
Eggs	152 (42.1)	126 (34.9)*	101 (48.1)	73 (34.8) *	51 (33.8)	53 (35.1)
Fish	216 (59.8)	205 (56.8)	132 (62.9)	124 (59.0)	84 (55.6)	81 (53.6)
White meat	299 (82.8)	278 (77.0)*	173 (82.4)	162 (77.1)	126 (83.4)	116 (76.8)
Red meat	85 (23.5)	59 (16.3)*	50 (23.8)	34 (16.2) *	35 (23.2)	25 (16.6)
Sweets	145 (40.2)	70 (19.4)*	84 (40.0)	39 (18.6) *	61 (40.4)	31 (20.5) *
	**All**		**BMI (kg/m^2^)**			
	**-**		**<25**		**≥25**	
	**N (%)**		**N (%)**		**N (%)**	
**Food group**	**Weekdays**	**Weekends**	**Weekdays**	**Weekends**	**Weekdays**	**Weekends**
Fruits	22 (6.1)	20 (5.5)	20 (6.3)	19 (6.0)	2 (4.5)	1 (2.3)
Vegetables	1 (0.3)	0 (0.0)	1 (0.3)	0 (0.0)	0 (0.0)	0 (0.0)
Cereals	58 (16.1)	54 (15.0)	49 (15.5)	46 (14.5)	9 (20.5)	8 (18.2)
Olive oil	18 (5.0)	17 (4.7)	17 (5.4)	15 (4.7)	1 (2.3)	2 (4.5)
Milk and dairy products	98 (27.1)	76 (21.1) *	89 (28.1)	69 (21.8) *	9 (20.5)	7 (15.9)
Nuts	31 (8.6)	28 (7.8)	29 (9.1)	27 (8.5)	2 (4.5)	1 (2.3)
Fermented beverages	355 (98.3)	335 (92.8) *	311 (98.1)	298 (94.0) *	44 (100.0)	37 (84.1) *
Potatoes	187 (51.8)	127 (35.2) *	170 (53.6)	115 (36.3) *	17 (38.6)	12 (27.3)
Legumes	151 (41.8)	89 (24.7) *	139 (43.8)	78 (24.6) *	12 (27.3)	11 (25.0)
Eggs	152 (42.1)	126 (34.9)*	133 (42.0)	107 (33.8) *	19 (43.2)	19 (43.2)
Fish	216 (59.8)	205 (56.8)	188 (59.3)	181 (57.1)	28 (63.6)	24 (54.5)
White meat	299 (82.8)	278 (77.0) *	263 (83.0)	242 (76.3) *	36 (81.8)	36 (81.8)
Red meat	85 (23.5)	59 (16.3) *	77 (24.3)	54 (17.0) *	8 (18.2)	5 (11.4)
Sweets	145 (40.2)	70 (19.4) *	127 (40.1)	60 (18.9) *	18 (40.9)	10 (22.7) *
	**All**		**Smoking status**			
	**-**		**No**		**Yes**	
	**N (%)**		**N (%)**		**N (%)**	
**Food group**	**Weekdays**	**Weekends**	**Weekdays**	**Weekends**	**Weekdays**	**Weekends**
Fruits	22 (6.1)	20 (5.5)	22 (6.7)	20 (6.1)	0 (0.0)	0 (0.0)
Vegetables	1 (0.3)	0 (0.0)	1 (0.3)	0 (0.0)	0 (0.0)	0 (0.0)
Cereals	58 (16.1)	54 (15.0)	54 (16.4)	52 (15.8)	4 (12.9)	2 (6.5)
Olive oil	18 (5.0)	17 (4.7)	16 (4.8)	15 (4.5)	2 (6.5)	2 (6.5)
Milk and dairy products	98 (27.1)	76 (21.1) *	88 (26.7)	71 (21.5) *	10 (32.3)	5 (16.1)
Nuts	31 (8.6)	28 (7.8)	29 (8.8)	27 (8.2)	2 (6.5)	1 (3.2)
Fermented beverages	355 (98.3)	335 (92.8) *	326 (98.8)	313 (94.8) *	29 (93.5)	22 (71.0) *
Potatoes	187 (51.8)	127 (35.2) *	176 (53.3)	121 (36.7) *	11 (35.5)	6 (19.4)
Legumes	151 (41.8)	89 (24.7) *	142 (43.0)	83 (25.2) *	9 (29.0)	6 (19.4)
Eggs	152 (42.1)	126 (34.9) *	136 (41.2)	114 (34.5)	16 (51.6)	12 (38.7)
Fish	216 (59.8)	205 (56.8)	199 (60.3)	187 (56.7)	17 (54.8)	18 (58.1)
White meat	299 (82.8)	278 (77.0) *	270 (81.8)	252 (76.4) *	29 (93.5)	26 (83.9)
Red meat	85 (23.5)	59 (16.3) *	80 (24.2)	57 (17.3) *	5 (16.1)	2 (6.5)
Sweets	145 (40.2)	70 (19.4) *	134 (40.6)	67 (20.3) *	11 (35.5)	3 (9.7) *
	**All**		**Physical activity status (minutes/week)**			
	**-**		**≥150**		**<150**	
	**N (%)**		**N (%)**		**N (%)**	
**Food group**	**Weekdays**	**Weekends**	**Weekdays**	**Weekends**	**Weekdays**	**Weekends**
Fruits	22 (6.1)	20 (5.5)	22 (7.9)	19 (6.8)	0 (0.0)	1 (1.2)
Vegetables	1 (0.3)	0 (0.0)	1 (0.4)	0 (0.0)	0 (0.0)	0 (0.0)
Cereals	58 (16.1)	54 (15.0)	52 (18.6)	47 (16.8)	6 (7.4)	7 (8.6)
Olive oil	18 (5.0)	17 (4.7)	16 (5.7)	12 (4.3)	2 (2.5)	5 (6.2)
Milk and dairy products	98 (27.1)	76 (21.1) *	84 (30.0)	66 (23.6) *	14 (17.3)	10 (12.3)
Nuts	31 (8.6)	28 (7.8)	27 (9.6)	25 (8.9)	4 (4.9)	3 (3.7)
Fermented beverages	355 (98.3)	335 (92.8) *	275 (98.2)	259 (92.5) *	80 (98.8)	76 (93.8)
Potatoes	187 (51.8)	127 (35.2) *	144 (51.4)	103 (36.8) *	43 (53.1)	24 (29.6) *
Legumes	151 (41.8)	89 (24.7) *	119 (42.5)	72 (25.7) *	32 (39.5)	17 (21.0) *
Eggs	152 (42.1)	126 (34.9) *	101 (36.1)	94 (33.6)	51 (63.0)	32 (39.5) *
Fish	216 (59.8)	205 (56.8)	171 (61.1)	161 (57.5)	45 (55.6)	44 (54.3)
White meat	299 (82.8)	278 (77.0) *	238 (85.0)	220 (78.6) *	61 (75.3)	58 (71.6)
Red meat	85 (23.5)	59 (16.3) *	69 (24.6)	47 (16.8) *	16 (19.8)	12 (14.8)
Sweets	145 (40.2)	70 (19.4) *	121 (43.2)	57 (20.4) *	24 (29.6)	13 (16.0) *

* Statistically significant differences. Evaluated by McNemar’s test.

**Table 8:** Responses of the study participants to the usability rating questionnaire for the e-12HR app.

	Questions, n (%)			
Options	Easy to Complete	Interesting to Complete	Understandable Questions	I Would Be Willing to Complete Again
Strongly agree	117 (67.2)	110 (63.2)	120 (69.0)	54 (31.0)
Agree	54 (31.0)	57 (32.7)	52 (29.9)	88 (50.6)
Neither agree nor disagree	3 (1.7)	6 (3.4)	2 (1.1)	28 (16.1)
Disagree	0 (0.0)	1 (0.6)	0 (0.0)	4 (2.3)
Strongly disagree	0 (0.0)	0 (0.0)	0 (0.0)	0 (0.0)
	**Questions, n (%)**			
**Options**	**Time to Complete the App**			
<1 min/day		16 (9.2)		
Approximately 1 min/day		37 (21.3)		
Approximately 2 min/day		63 (36.2)		
Approximately 3 min/day		43 (24.7)		
Approximately 4 min/day		11 (6.3)		
5 min/day or more		4 (2.3)		

## Data Availability

The data presented in this study are available on request from the corresponding author.

## Appendix A

**Table A1:** Questionnaire used in e-12HR.

**Questions About Food Groups:**
1. How many servings of fruits (orange, apple, pear, peach, strawberry, watermelon, etc., including fresh juice) have you consumed today? 1 serving = 150–200 g. Homemade measures: 1 serving = A medium-sized piece of apple, pear or orange, a cup of cherries or strawberries, two slices of melon, a glass of natural juice.
2. How many servings of vegetables (tomato, carrot, bell pepper, lettuce, zucchini, etc.) have you consumed today? 1 serving = 150–250 g. Homemade measures: 1 serving = One normal single plate of salad, one normal single plate of cooked vegetables, one large tomato, two carrots.
3. How many servings of breakfast cereals have you consumed today? 1 serving = 20–30 g. Homemade measures: 1 serving = One individual normal bowl.
4. How many servings of pasta have you consumed today? 1 serving = 50–70 g. Homemade measures: 1 serving = One normal individual plate.
5. How many servings of rice have you consumed today? 1 serving = 50–70 g. Homemade measures: 1 serving = One normal individual plate.
6. How many servings of bread have you consumed today? 1 serving = 30–60 g. Homemade measures: 1 serving = Three or four slices of bread or a muffin.
7. How many servings of olive oil have you consumed today (used for salad, to add to bread or for cooking)? 1 serving = 15 mL. Homemade measures: 1 serving = One tablespoon.
8. How many servings of milk and dairy products (yogurt, fresh cheese, aged cheese) have you consumed today? 1 serving of milk = 200–250 mL. 1 serving of yogurt = 125 g. 1 serving of fresh cheese = 60–80 g. 1 serving of aged cheese = 30–40 g. Homemade measures: 1 serving = A glass of milk, a yogurt, a tub or individual portion of fresh cheese, two or three slices of aged cheese.
9. How many servings of nuts (almonds, walnuts, hazelnuts, etc.) and/or olives have you consumed today? 1 serving = 20–30 g. Homemade measures: 1 serving = A handful of olives (8–10 units), a handful of hazelnuts (18-20 units), three or four walnuts.
10. How many servings of beer have you consumed today? 1 serving = 200 mL. Homemade measures: 1 serving = A beer or a bottle (200 mL). 1.5 servings = A bottle (large bottle), a can or a large beer glass (330 mL).
11. How many servings of wine have you consumed today? 1 serving = 100 mL. Homemade measures: 1 serving = A glass of wine (100 mL).
12. How many servings of potatoes have you consumed today (cooked, roasted or fried)? 1 serving = 100–150 g. Homemade measures: 1 serving = One large potato or two small potatoes.
13. How many servings of legumes (lentils, beans, chickpeas, peas, etc.) have you consumed today? 1 serving = 50–70 g. Homemade measures: 1 serving = One normal individual plate.
14. How many servings of eggs have you consumed today? 1 serving = One medium egg (50–70 g).
15. How many servings of fish (and / or shellfish) have you consumed today? 1 serving = 100–150 g. Homemade measures: 1 serving = One single regular steak. 0.5 servings = One can of tuna, etc.
16. How many servings of white meat (poultry) have you consumed today? 1 serving = 100–125 g. Homemade measures: 1 serving = One regular single filet or a chicken leg quarter. 0.5 servings = Two or three slices of chicken or turkey.
17. How many servings of red meat (beef, pork or lamb) have you consumed today? 1 serving = 100–125 g. Homemade measures: 1 serving = One single regular steak.
18. How many servings of processed meats (hamburgers, sausages) and/or cold cuts (salami, chorizo, cooked ham) have you consumed today? 1 serving = 90–100 g. Homemade measures: 1 serving = a hamburger, a large sausage or two small sausages, eight or ten thin slices of cold cuts (salami, chorizo), four or five thin slices of cooked ham.
19. How many sweets (sugar, candies, pastries, sweetened fruit juices and soft drinks) have you consumed today? Consider the number of units consumed regardless of the serving size.
**Additional questions about physical activities:**
20. For how many minutes have you practiced moderate physical activity today? Consider only those moderate physical activities that you practiced for at least 10 min at a time. According to the WHO, these activities require moderate effort, which perceptibly accelerates the heart rate. Examples: fast walking, cycling, dancing, housework, active participation in games and sports with children, etc.
21. For how many minutes have you practiced intense physical activity today? Consider only those intense physical activities that you practiced for at least 10 min at a time. According to the WHO, these activities require a great deal of effort and cause rapid breathing and a substantial increase in heart rate. Examples: running, cycling fast, competitive sports and games, etc.

**Table A2:** Usability rating questionnaire for e-12HR.

**1. SI found e-12HR easy to complete:**
Strongly agree. Agree. Neither agree nor disagree. Disagree. Strongly disagree.
**2. I found e-12HR interesting to complete:**
Strongly agree. Agree. Neither agree nor disagree. Disagree. Strongly disagree.
**3. I found the questions of e-12HR understandable:**
Strongly agree. Agree. Neither agree nor disagree. Disagree. Strongly disagree.
**4. In the future, I would be willing to complete e-12HR again:**
Strongly agree. Agree. Neither agree nor disagree. Disagree. Strongly disagree.
**5. How much time was needed to complete the daily questionnaire on the app:**
Less than 1 min per day. Approximately 1 min per day. Approximately 2 min per day. Approximately 3 min per day. Approximately 4 min per day. 5 min per day or more.

**Table A3:** Mediterranean diet Serving Scores (MDSS).

Food Group	Recommendation	Score
Fruits	1–2 servings/main meal	3
Vegetables	≥2 servings/main meal	3
Cereals	1–2 servings/main meal	3
Olive oil	1 serving/main meal	3
Milk and dairy products	2 servings/day	2
Nuts	1–2 servings/day	2
Fermented beverages	1–2 glass/day	1
Potatoes	≤3 servings/week	1
Legumes	≥2 servings/week	1
Eggs	2–4 servings/week	1
Fish	≥2 servings/week	1
White meat	2 servings/week	1
Red meat	<2 servings/week	1
Sweets	≤2 servings/week	1
Total maximum score		24

Main meal: breakfast, lunch and dinner. Cereals: breakfast cereals, pasta, rice and bread. Olive oil: used on salads or bread or for frying. Milk and dairy products: milk, yogurt and cheese. Fermented beverages: wine and beer (one glass for females and two glasses for males). White meat: poultry. Red meat: pork, beef and lamb. Sweets: sugar, candies, pastries, sweetened fruit juices and soft drinks.
